# Correction of severe columella and tip retraction in silicone implanted Asian short noses

**DOI:** 10.1186/s40463-016-0131-9

**Published:** 2016-03-10

**Authors:** Serkan Sertel, Ioana Irina Venara-Vulpe, Philippe Pasche

**Affiliations:** Department of Otorhinolaryngology, Head & Neck Surgery, University Hospital CHUV, Rue du Bugnon 46, 1011 Lausanne, Switzerland; Department of Otorhinolaryngology, Head & Neck Surgery, University of Heidelberg, Heidelberg, Germany

**Keywords:** Augmentation rhinoplasty, Nasal lengthening, Silicone implant, Gingivobuccal flap, L-shaped rib cartilage graft, Short nose, Anthropometric measurement

## Abstract

**Background:**

Silicone Implants and other alloplastic materials are frequently used in rhinoplasty to augment Asian short noses. However, nasal deformities as a result of implant-related infections are increasing in incidence. The resulting tissue scarrings hinder the application of traditional techniques of lengthening short noses. The following paper presents a technique to correct severe postoperative retractions of the tip and columella caused by silicone implants.

**Methods:**

We present a retrospective case study of two Asian patients with recurrent acute infections, secondary to silicone dorsum implants, leading to chronic inflammation of the tip and columella. The treatment consisted of implant removal and the immediate nasal reconstruction by combining uni- or bilateral gingivobuccal flaps along with L-shaped costal cartilage grafting.

To evaluate the surgical results, various anthropometric measurements, particularly the nasal length (NL) and nasal tip projection (NTP) of pre- and postoperative profile photographs, were analyzed.

**Results:**

Successful nasal lengthening and correction of columellar retraction were achieved. In case I, postoperative NTP and NL increased by 34.7 % and 21.1 %, respectively. In case II, NL and NTP increased by 23.8 % and 10.6 %, respectively. However, case II presented necrosis of the distal extremity of one gingivobuccal flap without rib graft resorption, which later healed by secondary intention.

**Conclusion:**

Pronounced columellar retraction in severe short noses can be successfully managed with a combination of gingivobuccal flaps along with L-shaped costal cartilage grafting. The use of autologous materials decreases the risk of long-term extrusion through the tip. The gingivobuccal flap provides vascularity to the exposed rib cartilage on the columella and prevents its resorption.

## Background

There is an increasing desire in Asian patients to have the same esthetical nasal features of Caucasians with a high and narrow nasal bridge, long columella and projected nasal tip. Rhinoplasty in Asian patients differs significantly from that of Caucasians. Thus, rhinoplasty in Asian patients mainly involves augmentation with grafts or implants, in contrast to resection, reduction, or refinement, which are typical for Caucasian patients. Nasal augmentation can be achieved with either autologous (bone or cartilage) or alloplastic material. Alloplastic materials such as silicone are frequently applied for dorsal augmentation rhinoplasty [1], due to their relative ease of insertion and the rapidity of the procedure.

However, due to implant-related complications subsequent removal of the silicone implant becomes often necessary [1, 2]. In fact, chronic inflammation and shifting of the implant in the subcutaneous tissue, resulting in a cephalic displacement towards the nasion and protrusion through the vestibular skin, are the most frequent problems after silicone implantation. The incidence of such complications is reported to be up to 36 % [1]. The consequences are skin contour deformities of the tip and columella, e.g. retraction and severe overrotation of the tip. The removal of the alloplastic material results in scar contracture of the dorsal and of the tip soft tissue [3].

The primary intentions of the reconstruction are to lengthen the overrotated nose and to correct the retracted columella. The latter is often challenging due to the lack of soft tissue in the membranous columella. First of all, a lack of tissue can be observed between the upper lateral cartilage (ULC) and the cephalic part of the lower lateral cartilage (LLC), which might not allow a caudal replacement of the tip. Moreover, soft tissue contracture along mucosal or external tissues can resist lengthening.

The applied lengthening techniques depend on the severity of the short nose. As Asian noses are generally shorter than Caucasian ones, a combination of maneuvers is necessary for their lengthening. Here, we present an innovative technique combining a wire-stabilized L-shaped rib cartilage graft with a uni- or bilateral gingivobuccal flap, which serves as a well-vascularized cover that also prevents the exposure of the L-shaped cartilage graft into the vestibule.

## Methods

This is a retrospective case study of two patients who underwent a revision augmentation rhinoplasty at the Centre Hospitalier Universitaire Vaudois (CHUV) in Lausanne. The Human Research Ethics Committee of the Canton Vaud approved the study protocol and the written informed consent.

### Case I: WM, ♀, * 16.06.1974

Initially the patient had an augmentation rhinoplasty with a silicone implant carried out in Thailand. Seven years later, she had a revision rhinoplasty because of a thickening of the right vestibule. Later she had recurrent infections of the right vestibule and the dorsum. In addition, she suffered from a renal insufficiency secondary to a glomerulonephritis and was listed for kidney transplantation. For this reason, it was decided to remove the silicone implant to prevent further infection considering her immunocompromised state. In the following years, she noticed an increasing retraction of the columella and infra-tip lobule as well as a synechia of the upper part of the right vestibule (Figs. 1 and 2). Laboratory findings could exclude Wegeners granulamatosis. Preoperatively, we administered a pathogen sensitive intravenous antibiotic treatment for five days, which was continued intraoperatively. During surgery new biopsies were collected around the silicone implant for an antibiogram to adapt the postoperative antibiotic therapy for 48 h intravenously and then for 15 days orally. The patient was followed-up 9 months after surgery, because she then moved back to Thailand.

**Fig. 1:**
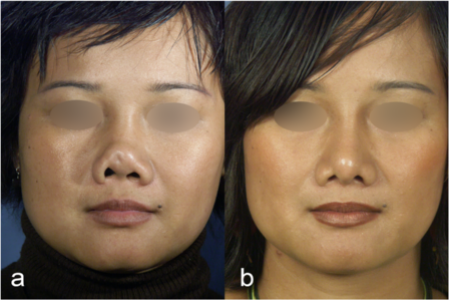
**a** Preoperative and **b** 9 months postoperative frontal views of case I

**Fig. 2:**
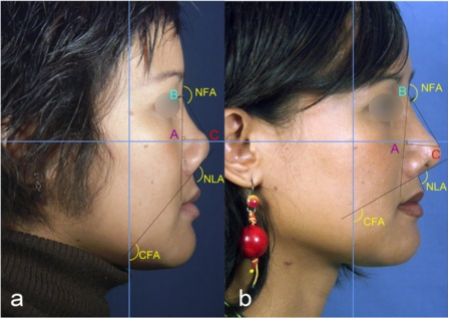
**a** Preoperative and **b** 9 months postoperative lateral views of case I with anthropometric measures. Reference points consisted of the tip-defining point (C), the nasion (B) and the projection of C onto the nasion-alar line (A). Nasal length (NL) was measured as the distance between B and C in centimeters (cm) according to the Goode’s method. Nasal tip projection (NTP) was measured as the distance from A to C in cm. The naso-frontal angle (NFA) was measured as the angle in degrees (°) formed between the proximal nasal dorsum and the anterior surface of the forehead below the glabella. The columellar-facial angle (CFA) was measured as the angle between the line drawn from the anterior columella to the subnasale and the line perpendicular to the Frankfurt horizontal plane (blue horizontal line). Postoperative NTP and NL increased by 34.7 % and 21.1 %, respectively

### Case II: SL, ♀, * 29.12.1961

This patient underwent an augmentation rhinoplasty with a silicone implant, followed by two revision rhinoplasties and finally the removal of the silicone implant because of a chronic infection. All operations were performed in Thailand. By the time she came to our clinic she had progressively developed a severe columellar retraction as well as a significant overrotation of the tip due to a lack of cartilaginous support. The distal border of the columella was severely retracted 3–4 mm behind the lateral part of the nostrils. Both soft triangles were also severely retracted, and synechia had reduced the height of the nostrils (Figs. 3 and 4). This patient was followed-up 3 years after surgery.

**Fig. 3:**
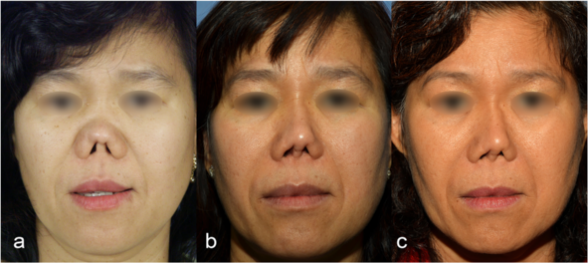
**a** Preoperative frontal view of case II with the columella severely retracted into the nose, **b** 8 months postoperative and (**c**) 3 years postoperative frontal views

**Fig. 4:**
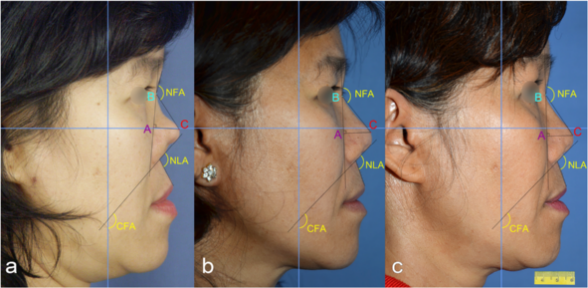
**a** Preoperative, **b** 8 months postoperative and (**c**) 3 years postoperative lateral views of case II with NL and NTP increased by 23.8 % and 10.6 %, respectively

### Operative techniques

#### Gingivobuccal flap

Several authors have described the transfer of the gingivobuccal flap into the nose [4–6]. In this case, its main indications were the closure of septal perforation and ozena. The flap was harvested according to Meyer’s method, without reinforcing the flap with ear cartilage [7, 8]. The vascularization of the gingivobuccal mucosa mainly depends on branches of the superior labial, infraorbital and buccal arteries [9]. In the case of a unilateral gingivobuccal flap, the pedicle is based 5 mm paramedian to the nasal spine. The main blood supply comes from the superior labial artery. However, when the gingivobuccal flap is harvested bilaterally, 2 cm of the untouched mucosa should be left between the two pedicles to preserve the vascularization of both flaps by the branches of the superior labial artery (R. septi nasi).

The flap was transferred to the nasal cavity through a tunnel next to the anterior nasal spine. The entire mucosa of the flap in the tunnel was also left intact. The two flaps covered the anterior septal cartilage on both sides, which in our cases consisted of the L-shaped rib cartilage graft (Figs. 5 and 6). The oral donor site was closed with resorbable sutures (Vicryl 4–0).

**Fig. 5:**
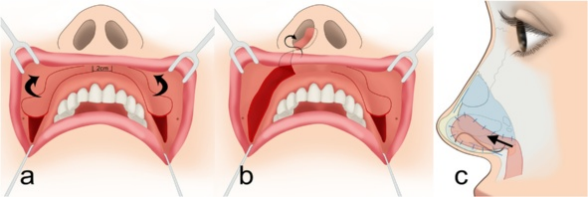
**a** Harvest of bilateral gingivobuccal flaps with 2 cm of untouched mucosa between the two pedicles in order to preserve vascularization of both flaps. Each flap is blood supplied by small midline branches of the superior labial artery. **b** Each flap is transferred into the nasal cavity through a tunnel beside the anterior nasal spine. **c** The two flaps cover the anterior septal cartilage on both sides (Modified drawing according to Meyer)

**Fig. 6:**
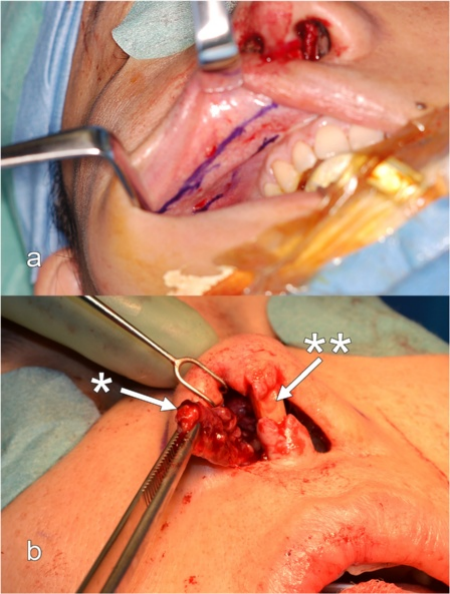
**a** Spoon-shaped gingivobuccal flap prepared from the mucosa and submucosa of the oral vestibule next to the frenulum above the upper row of teeth. The superior incision of the flap is done inferior to the stenon duct. **b** Flap passed through an oronasal tunnel into the nose (* Gingivobuccal flap; ** L-shaped graft)

### L-shaped rib cartilage graft

Severe short noses require an augmentation of the dorsum as well as the retracted columella projection and show. The donor site for the L-shaped costal cartilage graft was the cartilaginous costal arch at the 8^th^ and the 9^th^ rib.[7] Modeling the shape of this graft is crucial for ideal and individual positioning and thus sticks to exact angles to the dorsum and the caudal septum. The L-shaped graft was carved out in the middle of the rib to avoid warping. A 10 mm wire was inserted in the middle of the horizontal and vertical part of the L-shaped graft to prevent long-term bending of the cartilage and to secure the fixation on the anterior nasal spine (Fig. 7b, c). The shape of the graft can be adapted precisely to the individual anatomy by performing a chondrotomy of the graft, which maintains its stability due to the wire (Fig. 7b).

**Fig. 7:**
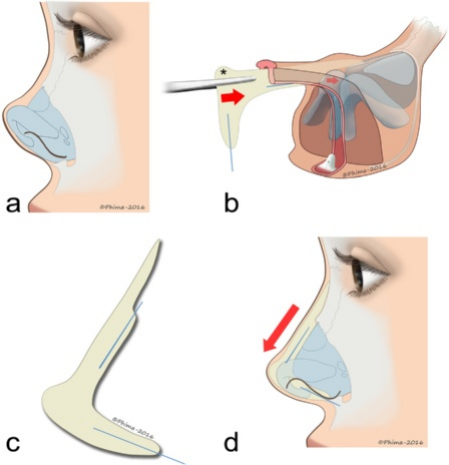
**a** Schematic aspect of a preoperative short nose. **b** L-shaped rib cartilage graft with caudal tip extension design (*), inserted with two wires into the cartilage to avoid its bending and to allow its fixation. Fixation with a vertical wire in a hole drilled into the nasal spine. **c** Design of the L-shaped graft with a notch to improve the stability over the dorsum. **d** Successful nasal lengthening and correction of columellar retraction

In order to relieve postoperative pain at the costal cartilage donor site we placed an intravenous canula and administered 5 ml of bupivacaine (2.5 mg/ml) right after closure of the wound and four hours postoperatively.

Patients were advised to do antiseptic mouth rinses with chlorhexidine for 10 days.

### Standardized photography

The patient photographs were taken in a standardized fashion with a digital single-lens-reflex (DSLR) camera (Nikon D5100) and a lens of 90 mm focal length by a professional photographer in the Department of Otorhinolaryngology, Head & Neck Surgery of CHUV. Patients were seated in a fixed position with a standardized distance of 1 m to the camera, and were asked to look at designated points for different views. The camera height was adjusted according to the patient’s height. Patients were asked to keep their eyes fully open with direct gaze and lips closed and not smiling.

### Anthropometric measurement

Photography analysis was performed using the Adobe^R^ Photoshop CS5 measuring tool (Adobe Systems, Inc., San Jose, CA, USA). Surgical results were analyzed by anthropometric measurements of pre- and postoperative profile photographs in the Frankfurt horizontal plane. Anthropometric measurements included the nasal length (NL), columellar-facial angle (CFA), nasal tip projection (NTP), naso-frontal angle (NFA) and the nasal tip projection ratio (Figs. 2 and 4). Reference points consisted of the tip-defining point (C), the nasion (B) and the projection of C onto the nasion-alar line (A) [10]. NL was measured as the distance in centimeters (cm) between B and C according to Goode’s method [11]. NFA was measured as the angle in degrees (°) formed between the proximal nasal dorsum and the anterior surface of the forehead below the glabella. NTP was measured as the distance in cm from A to C. The CFA was measured as the angle between a line drawn from the anterior columella to the subnasale and the line perpendicular to the Frankfurt horizontal plane. The nasal tip projection was determined according to Goode’s index [11] using the ratio: AC/BC. All anthropometric values were listed and compared pre- and postoperatively, expressed in differences (∆) and percentage differences (∆ [%]) of different time intervals (Table 1 and 2).

**Table 1:** Anthropometric values (Nasal tip projection (NTP), Nasal length (NL), NTP ratio equals NTP / NL, Columellar-facial angle (CFA), Naso-frontal angle (NFA)) of preoperative and postoperative results as well as their differences (∆) and percentage differences (∆ [%]) of case I

		NTP (cm)	NL (cm)	NTP ratio	NFA (°)	CFA (°)	NLA (°)
Pre-OP		1.9	3.64	0.52	145	140.5	118
Post-OP (9 months)		2.56	4.41	0.58	139.7	119.8	98.5
9 months post-op	∆	+0.66	+0.77	+0.06	- 5.3	- 20.7	- 19.5
	∆ [%]	+34.73	+21.15	+11.53	- 3.65	- 14.73	- 16.52

**Table 2:** Anthropometric values of preoperative, early (8 months) and late (3 years) postoperative results as well as their differences (∆) and percentage differences (∆ [%]) of case II

		NTP (cm)	NL (cm)	NTP ratio	NFA (°)	CFA (°)	NLA (°)
Pre-OP		1.6	2.68	0.59	134	147.3	121.6
Post-OP (8 months)		1.96	3.03	0.64	132.1	140	124.8
Post-OP (3 years)		1.77	3.32	0.53	135.7	136.2	116.3
8 months post-op	∆	+0.36	+0.35	+0.05	- 1.9	- 7.3	+3.2
	∆ [%]	+22.5	+13.05	+8.47	- 1.42	- 4.95	+2.63
3 years post-op	∆	+0.17	+0.64	- 0.06	+1.7	- 11.1	- 5.3
	∆ [%]	+10.6	+23.88	- 10.17	+1.26	- 7.54	- 4.36

## Results

### Surgical management

#### Case I (WM, ♀, *16.06.1974)

We performed an open rhinoplasty to remove the silicone implant. Chronic infection had destroyed the anterior septum and severely damaged the ULC and LLC. The right vestibular skin was destroyed with granulation tissue over a distance of 1.5 cm. The implant was displaced laterally towards the tip and protruded through the right vestibular skin (Fig. 1a). The silicone implant was removed. The previous implant site was cleaned of granulation tissue with a curette and then irrigated with a betadine/saline solution. Extensive subperiostal undermining of the nasal skin up to the nasion and the anterior wall of the maxilla was performed, in order to maximally mobilize the skin envelope. In addition, scarred tissue between the ULC and LLC was removed for mobilize the tip downwards. Despite mobilizing the septo-mucoperichondral flaps, a gap of 1.5 cm was observed in the right vestibule that was covered with a right gingivobuccal flap (Fig. 6). A spoon-shaped flap from the mucous membrane of the oral vestibule, next to the frenulum above the upper row of teeth was prepared and then passed into the nose through an oronasal tunnel (Fig. 5) [7, 8]. The flap was long enough to add mucosal tissue between the ULC and LLC. A sponge soaked with betadine was left in place in the previous silicone site during the preparation of the graft.

Cartilage was harvested from the 8^th^ rib and carved to an L-shaped form with a tip extension to recreate the dome according to the form of the silicone implant. The remaining LLC were sutured to the tip.

Two wires were inserted into the graft to avoid bending of the cartilage and to fixate the rib graft in a hole drilled into the anterior nasal spine. For the dorsum, a notch was created on the cartilage that was embedded into the nasal bone with an extension over the nasal bone (Fig. 7b–d). The second wire can also be placed under the nasal bone to support the stability of the fixation but it is not mandatory. The well-vascularized gingivobuccal flap covered the graft and additionally avoided exposure of the L-shaped cartilage into the vestibule.

#### Case II (LS, ♀, *29.12.1961)

The same technique as described in case I was used. The main difference was the pronounced skin retraction in the columella-dorso-tip unit, which allowed minimal downward mobilization despite maximal undermining of the skin. The columella was retracted into the nose behind the nostrils. Additional lengthening of the nose would have required a paramedian forehead flap, which was initially refused by the patient. During our first surgery, we removed the scarred tissue between ULC and LLC and replaced it with an auricular composite graft. The reconstruction of the membranous columella and the vestibule required a bilateral gingivobuccal flap. The L-shaped rib cartilage was inserted as in case I. Ten days later the patient developed a limited distal necrosis of the left gingivobuccal flap without resorption of the rib graft, which later healed by secondary intention. During this time, a minor tip deviation to the left was noted. Within 5 months we performed a reduction of the contralateral flap to improve the nasal breathing.

### Anthropometric measurement

#### Case I

The postoperative NTP increased by 34.7 % from 1.9 cm to 2.56 cm within 9 months. Nasal length (NL) increased by 21 % from 3.64 cm to 4.41 cm. NTP ratio according to Goode’s method increased by 11.5 %. All measured angles, NFA, CFA and NLA decreased postoperatively by 3.6 %, 14.7 % and 16.5 %, respectively (Table 1).

#### Case II

The first postoperative values of NL, NTP and NTP ratio, measured after 8 months increased by 13 %, 22.5 % and 8.5 % respectively. NFA and CFA decreased postoperatively by 1.4 % and 4.9 %, respectively. In contrast, NLA increased by 2.6 % (Table 2). The second postoperative measurement after 3 years revealed an increase of NL and NTP of 23.8 % and 10.6 %, respectively. Within the same time period, NTP ratio decreased by 10 %. NFA increased by 1.2 %, whereas CFA and NLA increased postoperatively by 7.5 % and 4.3 %, respectively (Table 2).

## Discussion

Augmentation rhinoplasty using alloplastic materials to correct short noses is a relatively common practice in Asia. Several materials are used for augmenting the height of the nose, e.g. silicone, Gore-Tex® and Medpor® [1]. However, alloplastic implant-related complications occur with an incidence of 4 % - 36 % [1, 12, 13] including infections, capsular contractures, extrusions, implant shifts, and calcifications.

The main problem in revision rhinoplasty after the extrusion of alloplastic-implanted material in short noses is the enormous scarring of the skin and inner lining, which hinders the application of traditional techniques. The skin can be mobilized by extensive undermining. If this is insufficient, a regional flap is an alternative. The disadvantage of the regional flap is scarring, which occurs even when nasal subunits are respected. This flap must cover the complete dorsal subunit as well as the dorsum and the tip subunit.

The surgical management of implant-related complications of short noses typically consists of two stages: (I) removal of the alloplastic implant and (II) reconstruction 10 days later in order to eradicate the infection and decrease the risk of cartilage graft infection. This approach however may lead to further scar contracture of skin and soft tissue during the time between the two stages. Therefore, we preferred simultaneous removal of the implant along with reconstruction, which was preceded by a pathogen sensitive intravenous antibiotic treatment.

After implant removal, we first needed to correct dorsum height and tip projection, which was initially achieved by the caudal part of the silicone implant. Secondly, we had to correct the overrotation by lengthening the entire nose. Finally, columellar and vestibular retraction by scarring required well-vascularized soft tissue replacement to cover the cartilaginous graft. All three problems were addressed with the L-shaped rib cartilage graft and the gingivobuccal flap. To maintain the soft tissue in the new position a strong stable support is required, therefore the anteriorly fixed L-shaped rib graft represents an ideal solution. However, the graft had to be fixed on the nasal spine with a wire. The second requirement for a stable reconstruction is the width of the columellar part of the L-shaped graft. It should be wide enough to present an adequate columella show. In addition, the L-shaped graft is maintained in position anteriorly and superiorly by close contact with the caudal septum. The shape of this graft is crucial for ideal and individual positioning and thus follows exact angles of the dorsum and the caudal septum.

With this technique, tip projection is augmented by a carved extension of the rib graft, similar to that of the initially created silicone implant (Fig. 7b).

Contemporary techniques used for nose lengthening include the extension spreader graft and the extented caudal septal graft, often combined with various tip and dorsal onlay grafts. Theses techniques require a strong septal support to maintain the grafts in the proper position. The severe short nose often presents a week septum due to an infection and extremely retracted skin, which can twist the grafts. We believe that the L-shaped graft provides a better stability of the reconstruction. Moreover, the tip extension in our L-shaped graft allows a higher fixation of the upper lateral cartilages and the creation of a new dome higher than the level of the previous domes. This assures a good projection with a uniform repartition of the pressure under the skin. In case of any resorption, the large amount of cartilage of the L-shaped graft can still preserve the stability of the reconstruction.

### Cases

Case I was successfully managed with optimal aesthetic and functional results. The gingivobuccal flap successfully covered the anterior septal cartilage and corrected the synechia of the upper part of the vestibule. Initially, the patient complained about nasal obstruction secondary to the thickness of the base of the flap. The problem resolved with a spontaneous atrophy of the flap after 6 months. Discomfort secondary to the scar in the gingivobuccal groove disappeared after 2 months of physiotherapy. No infection occurred despite the fact she was immunosuppressed in the context of kidney transplantation. A follow-up of 9 years showed a stable result with no long-term complications.

Anthropometric measurements confirmed the subjective aesthetical improvements. NL and NTP increased by 21.1 % and 34.7 %, respectively. The main reason for this improvement is rooted in the stability of the reconstructed cartilaginous framework with wires and the presence of individual skin elasticity, which allowed lengthening.

The unilateral gingivobuccal flap proved to be safe and very useful to replace the vestibular inner lining both on the medial side and the upper lateral wall of the nostril.

The length of nose in case II still maintained after a follow-up of 3 years. Comparison of the first with the last (3 years post-op) measurements revealed, that NL and NTP even increased by 23.8 % and 10.6 %, respectively. Despite wide subperiostal skin elevation, mobilization of the severely scarred skin envelope was limited. Additional lengthening would have required a regional flap, e.g. paramedian forehead flap to reconstruct the entire columella-dorso-tip or at least the dorsum unit. Unfortunately, the patient refused the paramedian forehead flap. Ultimately, the patient developed a limited distal necrosis of the left gingivobuccal flap without rib graft resorption that healed by secondary intention. This probably caused a minor tip deviation to the left. Debulking of the contralateral flap was performed after 5 months to improve nasal breathing. Major improvements were observed only on the tip, infra-tip and columella, which showed better volume, contour and definition in the frontal view (Fig. 3).

In such cases of severe short noses, we usually observe a deficiency of inner lining of the membranous septum as well as between the ULC and LLC. In addition, scarring of the vestibule reduces the height of the nostril. An elevation of a large septo-muco-perichondrial flap would lengthen the nose to maximally 4 mm anteriorly. An anteriorly based septal flap would add an anterior lengthening with the disadvantage of crusting of a denuded posterior septum. Composite conchal grafts represent a reasonable additional method to add tissue between ULC and LLC. These grafts should only be placed in a well-vascularized surrounding tissue. In case II, we successfully integrated one composite graft between the right ULC and LLC.

In our opinion, the gingivobuccal flap is useful for the reconstruction of the membranous septum. This flap was previously described for septal perforations and was used in three stages [7]. The question arises whether a bilateral gingivobuccal flap is advised. This approach bears the risk to lose one of both. The median sublabial region must be respected on a distance of 2 cm in order to protect the branches of the superior labial artery (Fig. 5a), which will vascularize both the flaps.

In cases of bilateral gingivobuccal flaps use, an autonomization of both flaps ten days before - as described in Meyer’s method to close septal perforations [8] - would probably decrease the risk of distal necrosis. However, this autonomization is not necessary in cases of unilateral usage of the gingivobuccal flap.

The major limitation of our technique is the confined mobilization of nasal skin envelope due to scar contractures.

The limitation of our study is the small number of patients.

## Conclusions

Pronounced columellar retraction in severe short noses can be successfully managed with a combination of gingivobuccal flaps and a L-shaped costal cartilage grafting. Stabilization and exact design of the L-shaped rib graft is the key to successful lengthening of severe short noses, because the contracted skin leads to high pressure on the graft with the risk of displacement. The use of autologous materials decreases the risk of long-term extrusion through the tip. The gingivobuccal flap provides vascularization to the exposed rib cartilage on the columella and prevents its resorption.

It is important to assess the status of the skin elasticity preoperatively. In these particular cases the mobilization of the nasal skin envelope will not be sufficient to lengthen the nose to satisfactory extent and a paramedian frontal flap should be taken into consideration.

## Consent

Written informed consent was obtained from the patient for the publication of this report and any accompanying images.
